# Lectures on Operative Dental Surgery and Therapeutics

**Published:** 1880-04

**Authors:** W. Finley Thompson


### ARTICLE III.

### *A Course of Lectures on Operative Dental Surgery and Therapeutics.*

BY W. FINLEY THOMPSON, M. D., D. D. S.

[Delivered at the National Dental College, London, 1879.]

No appliance in the operating room requires more careful attention than the dental engine, and the profession have become so wedded to its use, that it is now almost indispensible to the well doing of their work. However, it is sometimes found in the worst possible condition—dilapidated, loose-jointed, and the hand piece so rusty, that an instrument can with difficulty be introduced or removed; but not alone to this is due the dread so often expressed by patients concerning it, for the frequently unskillful manner of using the engine, has also caused it to become still more unpopular. Try to keep your engine in the very best working order; the bearings well oiled, free from a gummy condition, and the flexible arm so positioned as to prevent too much play. Use no more oil on the hand piece than is absolutely necessary, for an excess will not only soil the fingers, but also the lips of the patient. Of the several engines now in use, the "White" seems to occupy a front position; the "Elliott" and "Morrison" are also well liked by many.

It is quite as important that attention be given to the instruments required, as to the engine itself, for a badly mounted disk may prevent the creditable finishing of your work. The burs and drills must be perfectly adapted to the socket, neither binding on introducing them, nor yet so loose as to give a clicking sound when pressed laterally to and fro. The revolving cylinder in the hand piece, should be carefully inspected, whether rotating smoothly and without oscillation; for if this vibratory movement occurs, the most delicate handling will not overcome the repeated rattling noise. Inspect your instruments before using, to see that none are bent, irregular in shape or roughly made, for if they do not rotate on their true centers, the hand piece will be speedily reduced to a condition of little value. After using them, they should be withdrawn from the hand piece and thrown into a receptacle, there to remain until the close of the day, then wiped with an oiled cloth, and placed in an instrument stand, ready for the coming day's operations. Attended to in this manner, they will not oxidize, and become rough upon their surfaces.

In the preparation of cavities, do not hold the instrument continuously for any length of time upon the tooth, neither let it revolve slowly. Use the engine under full motion, directing the instrument with true precision to the portion of tooth to be operated upon; then with firm but delicate pressure, and quick alternate applications, continue the work as rapidly as possible until finished, being careful to give time between the alternations to prevent great thermal change.

Should danger exist from rapid execution, the engine ought to be displaced by the hand instrument. It is important to observe these principles when working near the pulp, as there can be no justifiable plea for exposing it in preparing the cavity; here your anatomical knowledge of the structures upon which you are working must save you from such an error.

Reviewing in detail the many individual appliances necessary for the operating room, the mallet deserves special mention. I have already spoken of it in connection with the preparation of cavities, but a more important consideration of its use is that of condensing the gold while filling. Here it becomes necessary to make a selection, there being different kinds, sizes and shapes, including the hand, electric, automatic and pneumatic mallets, all of which have their firm advocates. I certainly do not wish to disprove the value of any of them, for I have seen undoubted work produced by each; so that the question resolves itself more into the manner of their being used, than the instruments employed. Among the hand mallets are those made of lead, of wood and of steel; of the first and last metals named, I, for a long time, failed to arrive at a satisfactory decision of permanent merit, each having advantages as well as objections. The wood, owing to its lightness, producing a jarring sensation, greatly complained of; and I never feel, in using it, that the filling is properly condensed, so that it has been abandoned by me for some time. The steel gives an elastic but noisy blow, causing the patient to imagine a great deal that is never really felt. On the other hand the lead produces less noise, with an entire absence of elasticity; and being apparently less objectionable than steel or wood, I have used it in preference to either of them.

Although dullness and inelasticity are properties so commonly associated with lead, these very seeming disqualifications may become in the hands of the dentist, a means to an end. The requisite elasticity is given by the method of holding the mallet in connection with the stroke from the hand and fingers, to be hereafter described. The dullness of the metal prevents a secondary rebound, and assures the operator that the blows are the result of actual calculation.

In advocating the mallet, I do not wish to deprecate the method of hand pressure, for in approximal cavities and upon labial surfaces, the combination of both will frequently be found to produce effective work. The mallet—whether electric, automatic, pneumatic or hand—should be most conservatively used, for if too great force is applied, or the blows unnecessarily prolonged, the gold to some extent loses its cohesiveness. Study if possible, to get an elastic blow, I mean by this, a rebound—without jar—as quickly as the instrument has been struck. The only way I have been able to obtain the nicely modulated and ever varying force required, has been by the use of the hand mallet.

This conclusion is the result of careful observation and experiment, intervening and extending over a period beyond the advent of the electric and automatic. I accord to the inventors of both these instruments, their adaptability to the condensation of gold, so far as anything in mechanics can apparently contribute; yet I can not but recognize the superiority of a blow intelligently struck, over that given mechanically. This perfect intelligence can only exist when the operator has the mallet in his own hand, for while the strokes from an assistant may approximate the requirements, it is impossible to operate with the same assurance, self-comfort and satisfaction, that would exist if the force to be employed were under individual control. The hand acting in concert with the brain, under circumstances capable of supplying the exact want, gives a "motor" that never fails to respond at the right moment, and this can only be relied upon when co-ordinated within oneself?

In the delicate manipulations rendered possible by the hand mallet, the operator may himself become subject to considerable nervous tension, in fact even to the éndangerment of his health, and the relief given at such a moment by the "motor" mallet can scarcely be overstated. In the process of long-continued building, when portions of gold, aggregating to considerable cubical dimensions, are to be consolidated, the advantage of applying an easily and conveniently directed power from an extraneous source is very great indeed, and has, no doubt contributed much to the advancement of dentistry considered as an art. The electric mallet is sometimes uncertain in its action, and I draw attention to this with the hope that a remedy will soon be found. In speaking of the hand mallet, a few prescribed rules regarding its use may be of service. The following are submitted for your consideration, viz:

1st.—Adaptability.

2nd.—Materials used for mallets.

3rd.—Manner of holding.

4th.—Execution.

Concerning adaptability, the size, shape, weight, form of handle, etc., should be decided upon, after which avoid any change, for the hand once schooled to any particular instrument, fails to respond in as ready a manner to any other.

Of the materials from which you may select, are lead, steel, wood, tin, rubber, etc. Of some of these, and of the different forms of mallet, I have already spoken.

The manner of holding the mallet is one of the important features connected with its use, and requires to be particularly studied. The handle should rest loosely between the thumb and index finger, assisted by the second finger, and its retention be quite independent of any other portion of the hand. With each stroke of the mallet, the handle should come in contact with the palm of the hand before reaching the instrument, thereby partially arresting the blow, preparatory to its return. I would not have you forget that the impetus to the stroke must be given from the wrist and hand only.

There is, however, no arbitrary or stereotyepd rule by which you can be governed in the use of the hand mallet. With this, as all other instruments used in operating about the mouth, study to attain a delicacy of touch that may be perceptible to your patients, for a heavy hand in dentistry is much to be dreaded.

The instruments used in filling teeth require delicately formed points, the serrations to be shallow, but sharp and clearly defined, that each pellet of gold may be well struck, securely fastened, and thoroughly blended with its predecessor, thereby losing its individuality in perfect union; otherwise there will be a sliding, uncertain action of the instrument while condensing the gold. A few instruments well selected will answer every purpose; more than are absolutely required, only cause confusion. The principal reason for having few instruments, is, that the hand becomes schooled in their use; and this familiarity enables you to operate with greater facility. It is, however, beyond my power to dictate the requirements of your individual wants.

Extensive paraphernalia in some cases shows a mistaking of the means for the end. In one of the comic periodicals there was lately given a series of pictures representing the efforts made by an artist to improve his materials; amongst other things was a golden palette and an easel of gigantic size, made of costly wood and of gothic design. On it was the one little picture which was stationary all the while these elaborate things were being produced. The artist inquired of a friend whether there was anything further wanted. "Nothing," was the reply, "but talent." If the friend did not add "industry" also, we will supply the deficiency. Talent is a great and enviable possession in dentistry as in other pursuits, but ability, the outgrowth of industry, eventually removes every obstruction to professional advancement.

Before dismissing the subject of instruments, I must draw your attention to those for hand pressure. These will be found of great value in the cervical walls of approximal cavities, and upon buccal margins where the cavities impinge upon the gum. Not only do the serrated ends of these differ from the last described in the angle they form with the axis of the instrument, but their proportions are much heavier, and the handles are of a shape convenient to obtain a firm and decided pressure.

In my second lecture I alluded to the necessary examination of the mouth, and also to the constitutional treatment of youth. I wish again to revert to the subjects, and to somewhat enlarge upon them. For in examining the mouth we endeavor to ascertain—

First, the density of the teeth and their fitness to receive gold as a filling.

Second, the applicability of plastic fillings to teeth not having sufficient density for gold.

Third, the proper management of children when brought for consultation.

Having so far completed our clinical examination of the mouth as to fully understand the condition of the teeth, our investigations are by no means at an end. We have to ascertain, as well as we can, the resisting power of the teeth to the destructive agencies in the mouth. Age will partly guide us in this, also the color of the caries, as well as resistance to the instrument in cutting.

Some teeth are hard and dense, and decay makes but slow progress in them; while others, deficient in the denser constituents of the tooth structure from imperfect development, becomes susceptible to every influence, whether local or constitutional. When teeth of this latter type are presented for treatment, it becomes a question of some importance to discriminate between the different materials that may be employed. Gold requires a good setting to insure permanency, and is not compatible with teeth incapable of resisting the slightest shock. Where you meet with teeth of this character—reduced by caries to mere blue transparent shells in a few months—they must be filled with a material that can be easily applied and often renewed, and in this way they may be saved for a considerable period; but your attention will be required at frequent intervals, to inspect the work so placed.

In the mouths of children, the use of plastic materials in permanent teeth is sometimes most essential, and serves an excellent purpose in carrying them along until an advance in age shall have so solidified the teeth, as to enable your efforts to be attended with greater success in the insertion of more permanent and useful fillings. Discrimination and judgment should be so nicely balanced as to produce an equitable weighing of facts; or gold as well as other materials will be brought into disrepute. Having ascertained that the tooth structure is of a character to permit gold being employed, it becomes your duty to use it—patient permitting—in the knowledge that gold only can be used as a permanent filling. One great difference between gold and plastic fillings is, that gold is not acted upon chemically by any of the tissues or secretions of the mouth, and in turn does not act chemically upon them. Its office is to mechanically close up the cavity, and so long as the tooth structure around it shall exclude the fluids of the mouth, this answers the purpose, in advance of any other material yet discovered.

This neutrality not being so absolutely preserved by other substances, we sometimes avail ourselves of their chemical reaction in a manner favorable to tooth preservation; and especially does this apply to tin. The use of this metal, for filling teeth, is of considerable antiquity, and the chief objection to it is the lack of resistance to attrition. For mal conditioned (soft and chalky) teeth, tin has a claim upon our consideration, owing to its specific action on the walls of the cavity it fills, resulting in a hardening process from oxidization, and from contact with them. Especially do I advocate tin for children's teeth when of this uncertain character, for I have seen the bad effects of using gold for operations of this class, and have witnessed the gradual disintegration of the tooth structure round the edges of the filling, extending far into the crucial interstices of the tooth. These teeth would be better preserved and more safely carried along to a period that would admit of gold, by the use of tin or some of the different plastic materials, and the little patient subjected to less punishment.

And now, concerning the management of children—as well as their teeth—when brought for consultation, I have to say, that you will have no class of patients more difficult to deal with. Not only is there the impatience of pain peculiar to early age, but the natural restlessness of child hood to contend with. The teeth of youth with their large pulps and imperfectly calcified dentine, are additionally susceptible of uneasiness. It is useless to attempt deception, the instrument may be concealed, fine representations made, or a cloud of delusive words used. The deception only lasts for a moment, the child is embittered against you, and becomes suspicious and even rebellious at each future occasion they are compelled to accept of your services. This prejudice stamped on the impressionable mind of the child, may unconsciously influence him in after life, and cause him to defer until the last moment, the most necessary dental operations.

The golden rule is, *never deceive.*

At this important period both parents and dentist should be in unison. Parental authority must be united with the kind, but really explanatory words of the operator. The higher feelings of the child, its fortitude and courage, must be appealed to, should the age permit. That some part of the operation will not cause pain, can be taught and demonstrated; while that part which will give pain, can be notified to the little patient, who will then nerve itself for the trial.

At no period of life do the teeth demand more attention than at the age of from six to twelve years. Parents should be taught that the teeth at this time of childhood should be examined repeatedly. In this six years the most important and peculiar changes in the mouth, take place. The deciduous teeth must not be rashly removed. Their presence, so to speak, encourages the development of the permanent teeth, and if the deciduous teeth be removed, the permanent ones may be retarded in their development, and also forced to make their appearance out of the proper arch. On the other hand, should the deciduous tooth be by any means prevented from occlusion, the obstruction may, in a similar manner, throw the permanent tooth out of line and cause disfigurement. So much of the health, happiness and sometimes the well-being in the world depends on the possession of a comely set of teeth, that no pains in regard to this should be spared.

It has been remarked that no set of organs have, in the aggregate, caused more pain than the teeth; without assenting to so sweeping an assertion, I may say that much of the pain might be averted by preventive measures. Parents should be impressed that although the deciduous teeth are ultimately to be replaced, yet some six years or more are required to complete the process, and that caries should be looked for and carefully guarded against in these teeth. The consequence of this would be the prevention of much pain to the little one, while nights of unbroken and ever needful sleep, now lost to child, parents and nurse, would be saved. Teeth ravaged by caries, or injured by unwonted applications, and by the untoward and irrepressible actions of childhood, with swollen and inflamed gums, give rise to considerable constitutional disturbances, and a lowness of condition favorable to the approach of serious diseases; or to reducing the powers of resistance should the diseases incidental to childhood, make their appearance.

The first appearance of the permanent teeth has always, to my mind, been deeply interesting; but not unfrequently they are a cause of anxious thought to parents, for at the moment of eruption, or as soon as the crown can be seen, we find that the seal of destruction—in many cases—has been set upon them. Especially is this the case with the six year old molars. For we see manifestations of disintegration, lines of imperfect development, the enamel and the dentine dis-united, and in fact find evidence showing us that the early loss of the tooth may be predicted.

The cause of this imperfect development is invariably constitutional; and it is in measures taken to fortify the general health and to aid in the nutrition of the body that a remedy for this distressing state of things, may be found. I am aware that these remarks belong to the therapeutical part of my lectures, and I shall have to recur to this again when I reach that part of the series; but I feel that this section would be incomplete were I not to mention the prophylactic measures necessary.

The ensurement of a general state of good health is of course a *sine qua non*, and the general measures taken with that view, do not enter within the scope of these lectures, but the development of the teeth is greatly aided by appropriate diet. In the preparation of flour from wheat, some of its most important constituents are eliminated, especially those which contain the greater part of the lime salts. This loss of material has been pointed out over and over again by physicians, as a loss to the nation. The diet of children should be guided with a special regard to the introduction of lime salts, so as to convey provision for the bony structures of the body—and amongst the supplicants for this pabulum are the teeth. Oatmeal, the coarser cereals, bread made of whole flour, brown bread and milk with lime water should enter largely into the dietary table of infancy, childhood and early youth. With insufficient nutrition, with defective assimilation, or with food improperly selected, rachitis, and kindred disorders are engendered in the frame, and the effect of each arrest of development painfully manifested in the teeth. As civilization advances, so will the necessity for the *mens sana in corpore sano* become more evident to the enlightened mind; and that the health and symmetry which is in the savage state and functional activity may have been ensured by the "survival of the fittest," will be aimed at by physiological and hygienic methods, while the aid of the specialist will be increasingly demanded.—*Monthly Review Dental Surgery.*

